# Impact of COVID-19 Pandemic on Ibero-American Urology Residents: Perspective of American Confederation of Urology (CAU)

**DOI:** 10.1590/S1677-5538.IBJU.2020.S120

**Published:** 2020-07-27

**Authors:** Nahuel Paesano, Fernando Santomil, Ignacio Tobia

**Affiliations:** 1 Federico Abete Hospital Malvinas Argentinas Department of Urology Buenos Aires Argentina Department of Urology, Federico Abete Hospital Malvinas Argentinas, Buenos Aires, Argentina; 2 Office of Residents and Young Urologists of the American Confederation of Urology Buenos Aires Argentina Office of Residents and Young Urologists of the American Confederation of Urology - CAU, Buenos Aires, Argentina; 3 Hospital Privado de Comunidad Mar del Plata Department of Urology Buenos Aires Argentina Department of Urology, Hospital Privado de Comunidad Mar del Plata, Buenos Aires, Argentina; 4 Hospital Italiano de Buenos Aires Department of Urology Buenos Aires Argentina Department of Urology. Hospital Italiano de Buenos Aires, Buenos Aires, Argentina

**Keywords:** Resistance Training, COVID-19 diagnostic testing [Supplementary Concept], Urology

## Abstract

**Introduction:**

Since World Health Organization (WHO) declared COVID-19 as a global pandemic, urology services have developed strategies to prioritize and not to differ urgent and oncological patient's medical attention, in order to optimize resources and decrease infection probability among staff and patients. This unprecedented situation has generated a decrease in assistance and academic activities in most medical residences. The aim of this manuscript is to evaluate the impact of this health crisis on training programs through a survey addressed to urology medical residents.

**Materials and Methods:**

Cross sectional designed study, with multiple-choice non validated survey answered online by residents. Questionnaire was developed through the CAU EDUCACION platform.

**Results:**

A total of 148 responses from 18 countries coming from Latin America and Spain answering the survey. Of total, 82% answered that the activity of their urology department was significantly reduced, attending only urgent surgical pathologies, 15 % that, the urology activity has been closed completely and the staff was assigned to COVID-19 patients care, 3% continue with the regular clinic activity. Likewise, 75% stated that their surgical training has been completely affected, 93% receive urological information through tools such as Skype, ZOOM meeting, Cisco Webex, being Webinar modality the most used. Despite technological boom, 65% answered their academic training has been partially or completely affected. Most of the surveyed residents consider that period of residence should be extended to retrieve the educational targets.

**Conclusion:**

This unprecedented reality is negatively impacting the heterogeneous residency programs that American Confederation of Urology (CAU) nucleates. It is necessary to continue with technological innovation and allocate time and resources to easily generate accessible tools to favor the training of future urologists.

## INTRODUCTION

Since World Health Organization (WHO) declared COVID-19 (SARS-Cov-2 Coronavirus) as a global pandemic on March 11, 2020, affected countries health systems have determined measures increasingly restrictive in order to contain population and decrease virus spread. In northern hemisphere countries, where contagion curve suffered an abrupt growth, urology departments started limiting their activity according to health system saturation.

Latin American countries had adopted preventive measures, postponing any activity that did not imply an emergency in surgical field to avoid health personnel and available beds occupation ( 1 ).

This behavior purpose is to increase sanitary capacity, increase anesthesiologists availability for acute respiratory crisis management, and avoid contagion among patients with elective urological pathologies. However, urology services developed new protocols to prioritize urgent patients with oncological pathologies care that cannot be deferred ( 2 ).

This unprecedented situation is significantly affecting the already heterogeneous residency programs in Urology American Confederation (Confederación Americana de Urología, CAU) area in terms of duration, continuous evaluation systems, accreditations and re-certifications, as well as with regard to training and access possibility to new surgical technologies and urological diagnosis ( 3 ).

Our objective is to evaluate the impact of the COVID-19 pandemic on academic and surgical training activity of residents in urology across Ibero-American countries that comprise CAU.

## MATERIALS AND METHODS

Cross sectional designed study, in which multiple-choice non validated survey with 10 closed questions in Spanish version was carried out, which were answered anonymously online by residents of different academic training years, using their mobile devices or personal computer.

Questionnaire was developed through the CAU EDUCACION platform and was distributed by social media and email in the period from April 23rd to April 29th, 2020. A simple descriptive analysis was carried out.

## RESULTS

**Figure 1:**
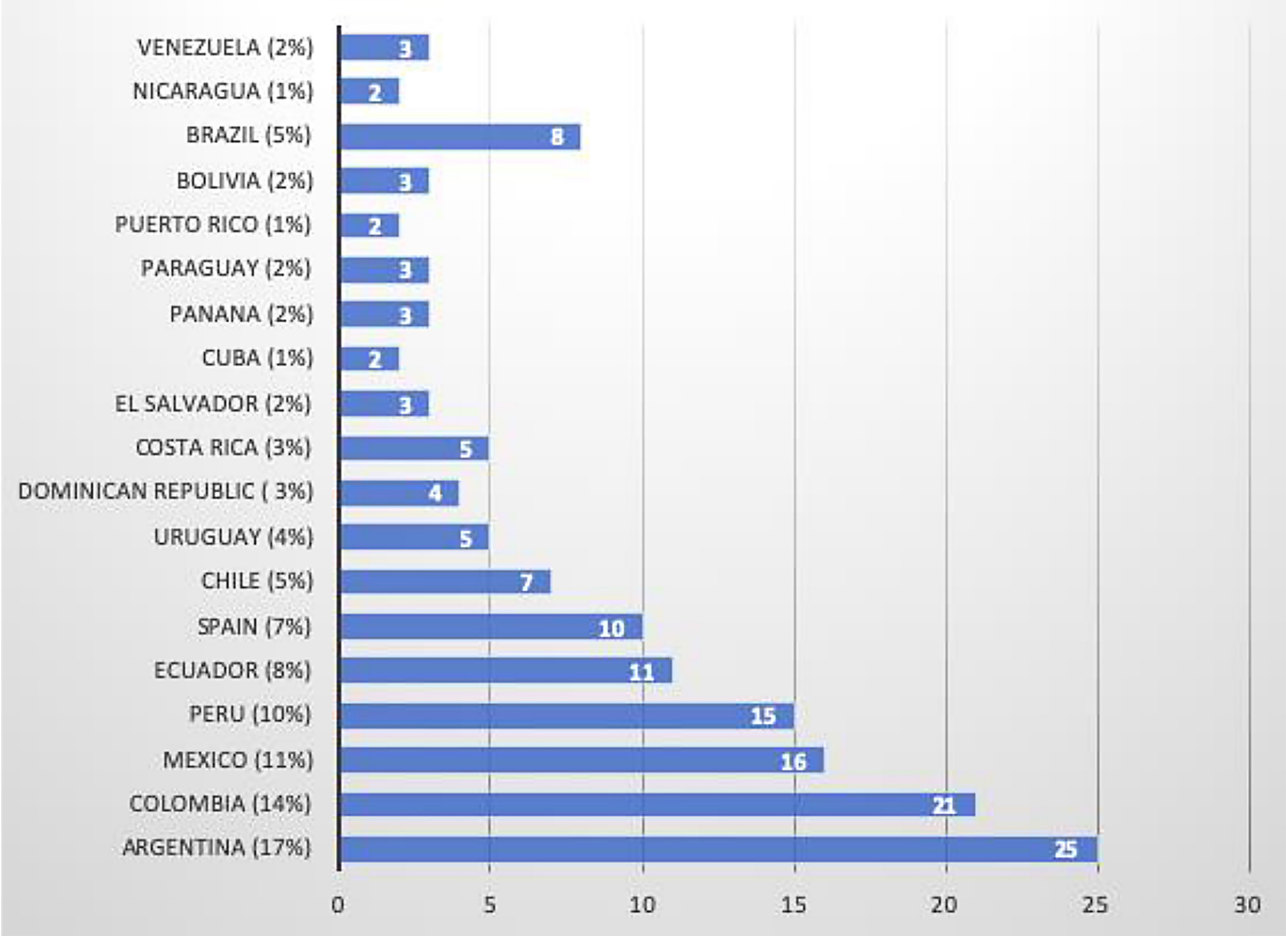
Report of percentages of responses by Country.

A total of 148 (100%) responses were obtained from medical residents of Argentina, Brazil, Bolivia, Chile, Colombia, Costa Rica, Cuba, Ecuador, El Salvador, Spain, Mexico, Nicaragua, Panama, Paraguay, Puerto Rico, Dominican Republic, Peru, Uruguay and Venezuela. ( Figure-1 shows the percentage of response by country).

A total of 24 (16.2%), were residents studying the first residence training year, 33 (22.3%) second, 34 (23%) third, 35 (23.6%) fourth and 22 (14.9%) fifth year.

Regarding urology services where residents carry out their activities current functioning, 121 residents (82%) reported that activity was significantly reduced, solving only urgent surgical pathologies such as testicular torsions, obstructive lithiasis, priapism, urological trauma, urosepsis or oncological diseases that could not be postponed according to main urological societies recommendation guidelines.

On the other hand, 23 (15%) responded their service has completely closed activity to dedicate itself to patients with suspected COVID-19 respiratory pathologies care. Only 4 respondents (3%) reported regular activity, attending to non-urgent pathologies patients, carrying out functional pathologies diagnostic studies and elective surgeries to correct urinary incontinence, benign prostatic hyperplasia, non-oncological penile scrotal pathology and lithiasis surgeries which do not require immediate resolution.

Of total, 134 residents (90%) have presented changes regarding their clinical activity. Workweeks are alternate, since they take turns with their colleagues to carry out basic administrative service work, they carry out patient's follow-up by telephone or telemedicine and they also collaborate with associate doctors in urological emergency surgery.

Only 9 (6%) are working exclusively in first line of care for patients with COVID-19 suspected respiratory pathology, while 5 (4%) are carrying out their usual tasks.

Main concern of residents surveyed is negative impact that this health crisis is generating on their surgical learning curve. Thus, 37 (25%) residents reported their activity in operating room has been partially interfered, while 111 (75%) stated their surgical training has been completely affected.

Regarding current academic resident's status, 138 (93%) acquire urological information through massive online dissemination tools with platforms such as Skype, ZOOM meeting, Cisco Webex, with Webinar (videoconference) modality being the most widely used, followed by pre-video edited surgeries, Journals clubs and Podcasts. Remaining 7% ( 10 ) do not carry out any online training type. But despite technological rise of these applications, 96 (65%) respondents affirm their theoretical training has been partially or completely affected, while the remaining 52 (35%) report that they have not undergone any change in their academic activity.

Due to this situation, 117 (80%) residents consider, based on their respective training programs, that measures should be taken being the most suggested to extend the residence period.

## DISCUSSION

In this COVID-19 outbreak scenario, a worrying residency program situation is evident, which aims to be a challenge both for trainers and for doctors in training, considering also uncertainty generated by not knowing this pandemic duration.

In addition to decrease in care and urology services training activities, clinical recommendations are that few non-deferrable surgical procedures that are performed be carried out by experienced doctors to reduce surgical times, risks of infection and complications ( 4 , 5 ).

Urological field has undergone gradual modifications according to alert level escalation in each country by COVID-19. In general, inter-hospital training instances were suspended, admissions exams to residencies were delayed, and face-to-face academic activities were suspended, beginning a new stage in scientific dissemination where applications such as ZOOM meeting, Skype and Webex Cisco play a critical role to interaction between residents and experienced physicians ( 6 , 7 ).

This unexpected period has provided an opportunity to explore different virtual learning options and should increase tools implementation such as telemedicine, smart training programs, and surgical skill development activities monitored by expert urologists ( 7 , 8 ).

From the CAU residents and young urologists office, scientific outreach programs have been successfully carried out through uro-oncological topics presentations through AULA VIRTUAL platform ( 9 ), as well as Ibero-American residents participation is encouraged through the contest “Camino a Guayaquil 2020”, which started in November 2019, and encourages all doctors continuous training within urological societies that comprise CAU, and it also promotes important scholarships obtainment.

Periodic virtual athenaeums development, clinical cases discussion, bibliographic reviews and surgical techniques through videos and simulation would provide a fundamental and complementary contribution ( 10 ).

Although these modalities do not replace learning process in operating rooms, they represent a challenge and encourage new educational technology strategies generation that could be incorporated in educational programs in the future.

Limitations of this study were the short disclosure time and the low number of responses with respect to the total number of urology residents in each country, and the strengths were the number of countries that participated that allowed giving a representative outlook on Spain and Latin America residents reality.

## CONCLUSIONS

This unprecedented reality has a negative impact on the heterogeneous residency programs at the American Confederation of Urology.

Main residents concern is focused on their surgical training.

Online modalities such as Webinar and Podcast are the most widely used and are currently a fundamental tool for continuous updating.

Most respondents suggest measures such as extending residency program to retrieve the educational targets.

We encourage entities responsible for training residents to continue with technological innovation and to allocate time and resources to generate easily accessible tools, such as surgical simulators, step-by-step videos of surgeries, and tutored surgical skills development programs tutored by experienced physicians to reduce the impact of this situation on learning curves and favor future urologists training.

## ABBREVIATIONS

CAU: American Urology Confederation
COVID-19: Coronavirus disease 2019
SARS-Cov-2 Coronavirus: Severe acute respiratory syndrome coronavirus 2
